# Oral manifestations of lymphoma: a systematic review

**DOI:** 10.3332/ecancer.2016.665

**Published:** 2016-08-17

**Authors:** Taísa Domingues Bernardes Silva, Camila Belo Tavares Ferreira, Gustavo Boehmer Leite, José Roberto de Menezes Pontes, Héliton S Antunes

**Affiliations:** 1 Instituto Nacional de Câncer (INCA), Rio de Janeiro, Brazil; 2 Library Section, Instituto Nacional de Câncer (INCA), Rio de Janeiro, Brazil; 3 Private practice; 4 Dentistry Section, Instituto Nacional de Câncer (INCA), Rio de Janeiro, Brazil; 5 Clinical Research Division, Instituto Nacional de Câncer (INCA), Rua André Cavalcante, n 37, 2 andar, Rio de Janeiro, RJ CEP20231-050, Brazil

**Keywords:** lymphoma, oral cavity, dental care, oral manifestations

## Abstract

Lymphoma is a malignant disease with two forms: Hodgkin’s lymphoma (HL) and non-Hodgkin’s lymphoma (NHL). Non-Hodgkin’s lymphoma is diagnosed in extranodal sites in 40% of cases, and the head and neck region is the second most affected, with an incidence of 11–33%, while HL has a very low incidence in extranodal sites (1–4%). The aim of this study was to identify the oral manifestations of lymphoma through a systematic literature review, which we conducted using the PubMed, Lilacs, Embase, and Cochrane Library databases. We found 1456 articles, from which we selected 73. Among the intraoral findings, the most frequent were ulcerations, pain, swelling, and tooth mobility, while the extraoral findings included facial asymmetry and cervical, submandibular, and submental lymphadenopathy. Among the few studies reporting imaging findings, the most cited lesions included hypodense lesions with diffuse boundaries, bone resorptions, and tooth displacements. The publications reviewed highlight gaps in the areas of early detection, diagnosis, and proper treatment.

## Introduction

Lymphoma is a heterogenous malignant disease of the lymphatic system, characterised by a proliferation of lymphoid cells or their precursors [1]. Lymphomas present different behaviours and degrees of aggressiveness and can be divided into two large groups: Hodgkin’s lymphoma (HL) and non-Hodgkin’s Lymphoma (NHL). Hodgkin’s lymphoma occurs mainly in the lymph nodes (>90%) [2] and only 1–4% of the cases involve extranodal areas [3–4], appearing as a nodal disease with predilection for neck and mediastinal nodes [2, 5–7]. Hodgkin’s lymphoma is diagnosed when the histopathological examination shows the presence of Reed-Sternberg cells [8] which are binucleate cells with a generally abundant cytoplasm and two large nucleoli (one in each core) that appear like ‘owl eyes’. Hodgkin’s lymphoma can be further classified as classic HL or lymphocyte-predominant HL with respective incidences of 95% and 5%. The former has a bimodal age distribution with an early peak in young people (ages 20–24) and another peak in elderly patients aged 80–84, while the latter can occur at any age, but most often occurs in individuals between 30 and 50 years of age [2]. The disease’s nodal evolution is contiguous, and its clinical evolution is slow and predictable which favours therapeutic protocols [3]. In extranodal site, NHL represents 40% of all lymphomas [2]. Over 20 different subtypes have been classified according to the type of lymphoid cell and its behaviour. Nodal evolution proceeds randomly with an unpredictable clinical outcome [3]. Morphological, immunophenotypic, and cytogenetic characteristics are essential for the classification of each subtype of NHL. The gastrointestinal tract is the most common site of extranodal disease, occurring in over 50% of patients, followed by head and neck which varies from 11–33% [9], where the most frequent age group is over 50 years old.

Lymphomas are the third most common cancer worldwide and constitute 3% of malignant tumours. They make up 2.2% of all malignancies of the head and neck and are the second most frequent in that region surpassed only by epithelial malignancies [10]. The global estimate of new yearly cases of NHL is 217,643 for males and 168,098 for females while that of HL is 38,520 for males and 24,430 for females [11].

Patients with acquired immunodeficiency syndrome (AIDS) have a higher risk to develop Non-Hodgkin’s lymphoma [12], about 100–200 times the risk of the general population [13]. This malignancy has been reported as the second most common in this group of patients, Kaposi’s sarcoma being the most common [13–14], and the extranodal presentation occuring more commonly in 70–80% of the cases [12]. In comparison with the general population, AIDS-related lymphomas have a rapid progression, bad response to the treatment, high relapse rates and overall poor prognosis [13 and 15].

The most recent and widely used classification is that of the World Health Organisation (WHO). It is based on the Revised European-American Lymphoma (REAL) classification and primarily subdivides lymphomas based on cellular origin: B cell lymphomas, T cell lymphomas, natural killer lymphomas, and Hodgkin’s lymphoma [16–18]. Furthermore, the WHO classification includes new immunogenetic, morphological, and molecular characteristics [19]. The most widely used lymphoma staging classification system is the Ann Arbor classification [20–22], which is based on the number of regions of the involved lymph nodes, and the presence of extranodal disease, disease above and below the diaphragm, and systemic symptoms [2].

The treatment of lymphoma in the head and neck is complex because of the numerous variables involved and depends on clinical staging [23]. The different treatment modalities include: 1) radiotherapy, which plays a limited role in the primary treatment of NHL and has been successful only when gingival lesions are present; 2) chemotherapy, with or without radiation, which is the modality most often used in most lymphomas and is generally recommended in disseminated disease stages III or IV; 3) growth factors that limit myelosuppression; and 4) bone marrow transplant and monoclonal antibodies which act against the surface antigens of affected cells [24].

Oral cavity lymphomas represent the third most common malignancy in the oral cavity, surpassed by squamous cell carcinoma and malignancies of the salivary glands. Lymphomas in the oral cavity are rare; only 3% of all lymphomas in the general population and 4% on patients with AIDS [25].

The manifestations of oral lymphomas are often difficult to diagnose because they present clinical features that mimic other diseases such as periodontal disease, osteomyelitis, and other malignancies [26]. This may delay the correct treatment thereby worsening the prognosis.

Thus, the aim of this systematic review was to identify the oral manifestations of lymphoma reported in scientific publications in order to assist health professionals in the initial diagnosis, prognosis, and treatment.

## Materials and methods

### Search strategy

We searched for articles published until 31 December 2015 in the following databases: Literatura Latino-Americana e do Caribe emCiências da Saúde (LILACS), Medline (via PubMed), Embase, and the Cochrane library (Cochrane reviews and Cochrane trials). Our search strategy involved combining terms associated with the inclusion and exclusion criteria as well as the guiding question in the different databases. We also used the Boolean operator not together with terms for treatment options in order to reduce the number of articles involving lymphoma treatments. We also restricted the search to studies performed with humans (Figure 1).

### Study selection criteria

Studies eligible for inclusion were: i) case reports, literature reviews, retrospective studies, or prospective studies that addressed the oral manifestations of lymphoma published until 31 December 2015; and ii) publications in English, Spanish, or Portuguese. Studies focusing solely on lymphoma therapies and outcomes, without information on the oral manifestations were excluded.

We found 153 publications in LILACS, 249 in Cochrane reviews, 179 in Cochrane trials, 447 in Embase, and 428 in PubMed.

**Figure 2.**
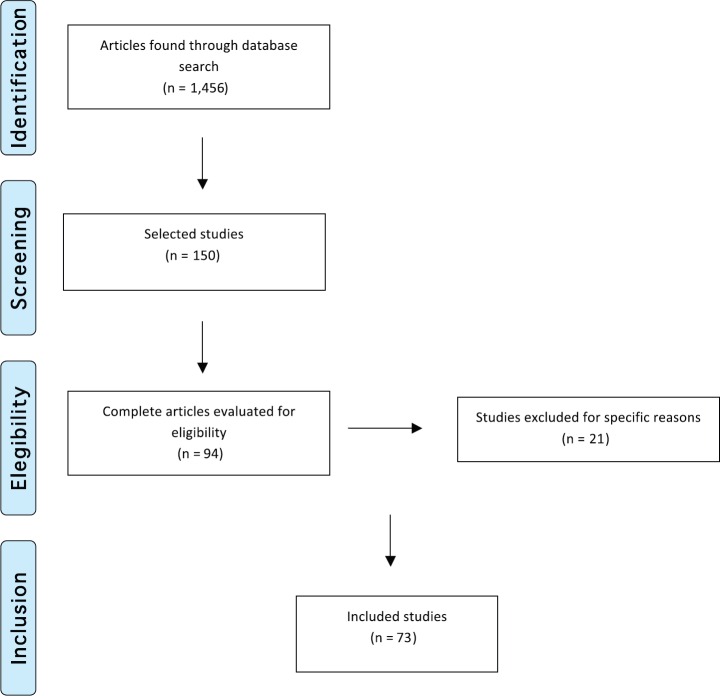
Flowchart illustrating study selection based on the PRISMA model (Adapted from Moher D et al (2009) The PRISMA Group, 2009). Preferred Reporting Items for Systematic Reviews and Meta-Analyses: The PRISMA Statement PLoS Med 6(6) e1000097. DOI: 10.1371/journal.pmed1000097)

We then conducted a second selection after reading the titles and abstracts. This selection left 150 references of which 94 remained for the following round. We then read the full text of the 94 studies and 21 of those were excluded for not meeting the established criteria. Thus, 73 studies made up the final selection as illustrated in the flowchart following the PRISMA model [27] shown in Figure 2.

### Data extraction

To extract data from the selected studies, we elaborated a document analysis chart containing the following variables: publication title, study authors, publication year, place of publication, and study design. For clinical case studies, the following variables were analysed: gender, age, type of lymphoma, lymphoma location, intraoral and extraoral examinations, and imaging studies.

## Results

The first article published on this topic was a case report from 1970. Of the 73 articles included in this systematic review, 51 were clinical cases (69.9%), 16 were retrospective studies (21.9%), four were narrative reviews (5.5%), and two was a systematic review (2.7%), including a total of 978 reviewed cases. Of these, 361 cases (36,9%) were reported in a single cohort study conducted in Canada by Epstein *et al* [10] and 87 cases (8.9%) were also reported in a single study, by Horiuchi *et al* [64] in the United States. These studies were responsible for reporting almost half of all cases.

In terms of lymphoma types, diffuse large B-cell non-Hodgkin’s lymphoma (DLBCL) was the most frequent, with 288 cases, followed by 99 cases of small cell non-Hodgkin’s lymphoma, and 79 cases of Burkitt’s lymphoma. All other types of lymphoma are listed in Table 1.

**Figure 3.**
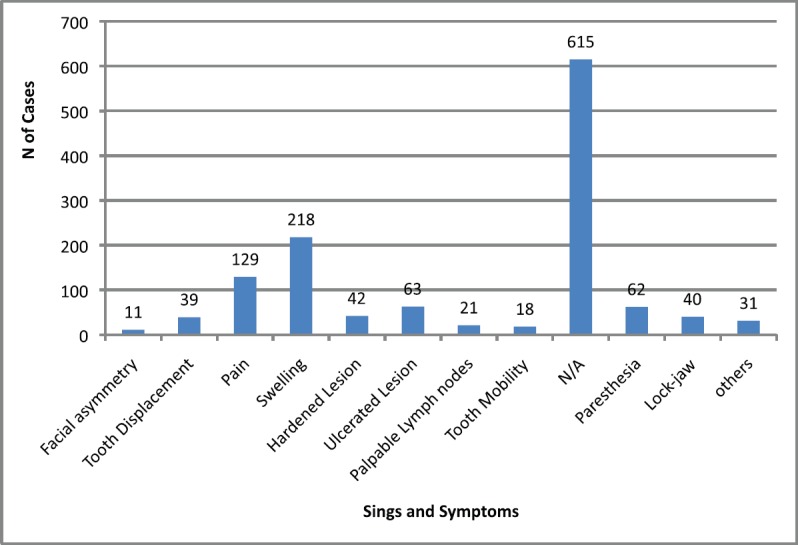
Signs and symptoms in head and neck region. Others = poor alveolar scarring, mandibular fracture, periodontal pocket, exposed bone, fistulas, bleeding, and macroglossia. N/A = not available.

**Figure 4.**
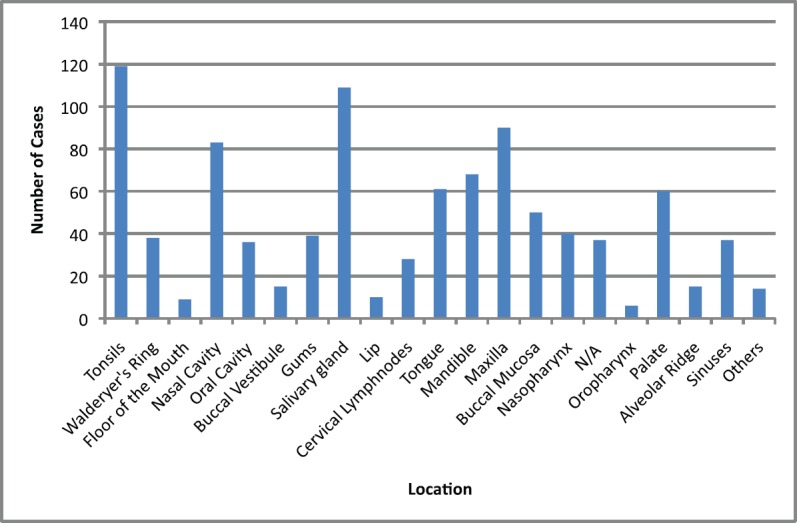
Location of lymphomas in head and neck region. Locations are listed as reported in the articles. N/A = not available; Others = lip commissure, zygomatic bone, masseter, and retromolar trigone.

**Figure 5.**
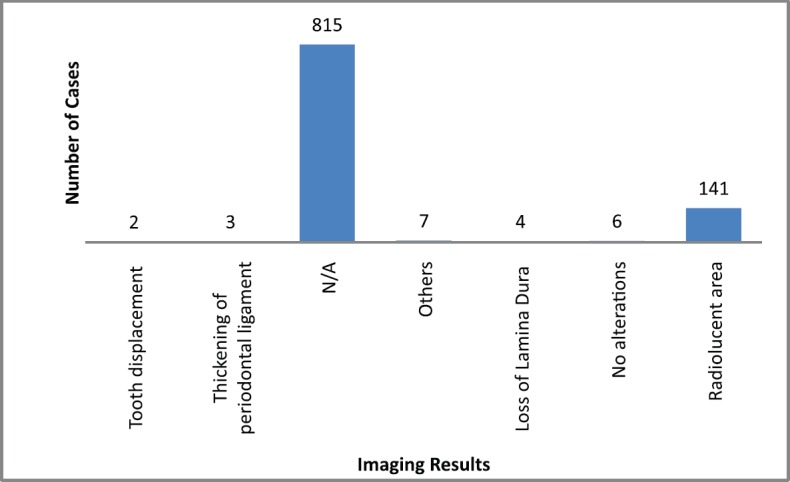
Imaging results. N/A = not available. Others = mandibular fracture, poor alveolar scarring, unclear image, image evoking invasive lesion, 6 cm increase, and infiltrative lesion.

The main clinical manifestations reported in the articles included in this systematic review were swelling in 218 cases, pain in 1289 cases, and paresthesia in 62 cases (Figure 3). As for location, the tonsils were the most affected in the head and neck region, appearing in 119 cases, followed by salivary glands with 109 cases, while the maxilla was the most affected bone region, with 90 cases reported (Figure 4). It is important to note that some articles did not describe the specific lesion sites and instead used broad terms such as ‘Waldeyer’s ring’ and ‘oral cavity’, which may not give a completely accurate idea of the most frequent location. Of the 163 cases with imaging results, the most frequent feature was the radiolucent image, meaning absence of bone or bone erosion, with 141 cases (Figure 5). As for gender, there were 546 males versus 432 females. The youngest patient was diagnosed with NHL at 2 years of age and the oldest at 96.

Of the clinical cases studied, 40.52% were initially misdiagnosed as a different pathology and were treated as such, delaying proper treatment, and allowing the progression of lymphoma. Most treatments were extractions, periodontal treatments, and abscess treatments. Many of the retrospective studies did not report the initial diagnosis of individual cases, precluding that evaluation.

## Discussion

This systematic review includes the presentation of 982 cases reported in 73 scientific papers, including 76 patients described in case reports papers and 906 patients presented in retrospective studies (Table 1). The articles were generated over the last 45 years which is when most studies on the subject were published, according to our search. The articles identified revealed a shortage of studies addressing the oral manifestations of lymphoma. The few studies addressing the oral manifestations presented few details about the intraoral clinical manifestations of lymphoma or radiographic findings. This shortcoming may be because of the challenging nature of studying and treating such patients.

Imaging is important for diagnostic confirmation and for better estimating prognosis, since the oral manifestations of lymphoma can mimic various diseases. There were no imaging results available for 815 cases of lymphoma in the head and neck region. This lack of imaging results made it difficult to compare and discuss radiographic features.

Among the 163 cases with radiological findings, most showed radiolucent images representing bone resorption or bone loss. Radiographically, intraosseous lesions appear as radiolucent areas both uni- and multilocular with diffuse edges [2]. Therefore, the radiographic manifestation observed in most cases could be a differential diagnosis of various bone diseases.

Some authors presented thickening of the periodontal ligament space [28, 34, 38, 46, and 70] and loss of the lamina dura [28, 34, and 46] as radiographic findings. The loss of the lamina dura in lymphoma patients may not simply be related to the effects of tumour cell infiltration. In some cases the bone changes in lymphoma patients may be because of the release of osteoclast-activating factors from the lymphoid cells [34]. These radiographic features can mimic various periodontal diseases such as periodontitis, periodontal abscess, and Papillon-Lefèvre syndrome, among others. In addition, the bone destruction observed in clinically aggressive lymphomas is indistinguishable from that of other malignant tumours of the jaw, [2 and 26] which reinforces the importance of biopsy for proper diagnosis.

In this review, we observed that diffuse large B-cell non-Hodgkin’s lymphoma (DLBCL) was the most common histological type of lymphoma in the head and neck region [2, 10, 53, 87–89]. Other authors add that it is the most common lymphoid malignancy among adults [90–93], representing 40% of lymphomas [2 and 94]. Extranodal DLBCL mostly affects older men in their seventh decade of life [80 and 95].

More than 90% of the AIDS-related NHL are B-cell origin. According to the WHO classification, two histological subtypes are dominant. DLBCL as the most frequent histological subtype, occurring in 70–80% of the cases and the Burkitt’s lymphoma (BL) representing 7–20% of all cases [96]. Plasmablastic lymphoma (PBL) is described as an aggressive and rare DLBCL subtype, commonly found in patients infected with HIV. It represents 2.6% of all AIDS-related lymphomas [97]. The Epstein-Barr virus is strongly associated to the pathogenesis of the PBL. It is found on 60–80% of the DLBCL and 50% of the Burkitt’s lymphoma [96]. The HHV-8 is another oncogenic virus that has been also related in association with PBL in patients with AIDS. The role of the HHV-8 on the pathogenesis of the PBL remains unclear but many authors have been reporting this fact. [98] The immunophenotype is similar to the myeloma because of the presence of plasma cell markers and the absence of B-cell markers [99].

PBL was originally described as a disease that involves the oral cavity of immunodeficient patients. Usually, it is associated with a poor prognosis and low survival rate after diagnosis, ranging from 4–11 months [99]. In the general population Waldeyer’s ring is the most common site of NHL of head and neck region. The tonsils are the most prevalent localisation. The AIDS-related NHL of the head and neck often presents as a large mass with bone destruction of the maxilla and sinuses. Inside the oral cavity, this large mass usually involves the gums and hard palate presenting as ulcerative lesions [13].

Burkitt’s lymphoma (BL) clinically occurs more often in children and is relatively rare in middle-aged or elderly adults [72]. Burkitt’s lymphoma can be classified into three clinical subtypes: the endemic type (African) which involves the jaws in over 50% of cases, the sporadic type which generally presents an abdominal mass [67], and the type associated with acquired immunodeficiency [72]. Several studies strongly suggest the involvement of the Epstein-Barr virus (EBV) in BL pathogenesis, since EBV inhibits programmed cell death and contributes to the development and maintenance of BL [60]. In this systematic review, BL was more common in males, and the most affected areas of this type of lymphoma were the mandible followed by the maxilla. The main findings in the intraoral and extraoral examinations were swelling, pain, dental displacements, and facial asymmetry. Regarding imaging, the main findings were bone resorption, followed by lesions with diffuse boundaries. According to Freitas *et al* [60], males are affected about twice as much as females, which is in line with the findings in this systematic review. To the best of our knowledge, there has been no satisfactory explanation for the common involvement of the jaws [34]. In the oral cavity, this tumour can progress rapidly and present itself as an exophytic mass or a facial swelling involving the jaws [100]. The tumour usually begins in the posterior maxilla and then spreads to the four quadrants, resulting in increased tooth mobility, intraoral masses, lip numbness, and tooth pain because of infiltration in the pulp, especially by developing teeth. Radiographically, it appears as noise traces with radiolucent edges, while cortical bone is expanded, eroded, or perforated by infiltration of soft tissues [101].

According to Colmenero *et al* [87], intraoral lymphomas’ first signs may appear as infection in 50% of the cases. Occasionally, oral manifestations may present as the first and only sign of disease [102], and dentists must provide the correct evaluation and proceed accordingly. Oral manifestations usually include asymptomatic soft swelling [26] with or without ulceration that primarily affect the tonsils, palate, buccal mucosa, gums, tongue, floor of the mouth, salivary glands, and retromolar region [102]. Alveolar bone loss with oedema and pain may also occur which often mimics periodontal diseases. This may lead to ineffective treatments with antibiotics and supra- and subgingival scaling as described in studies presented by Spatafore *et al* [38], Dood *et al* [40], and Nittayamanta *et al* [46], where incorrect treatment further delayed the onset of lymphoma treatment, worsening the prognosis. Authors such as Richards *et al* [26], Forman *et al* [28], Groot *et al* [39], Rosenberg *et al* [44], Freitas *et al* [60] and Santos *et al* [62] presented clinical cases in which extractions were conducted simply because there was tooth mobility and periodontal pockets, or because of previous unsuccessful periodontal treatment or pericoronaritis. Lip paresthesia and pathologic fractures may also occur, and are common signs of jaw involvement [90]. Some authors such as Wright *et al* [30], Cohen *et al* [32], Conteras *et al* [50], Farias *et al* [59], and Bombeccari *et al* [69] made an initial diagnosis of traumatic injury because of the presence of trauma prior to swelling presented at the time of clinical examination. This swelling caused these authors to opt for incisional biopsy in an effort to aid in appropriately beginning the treatment. Some patients may also have regional lymph node involvement [73], which in most cases is initially diagnosed as tonsillitis and treated with antibiotics, as noted in the study by Chicareli *et al* [71]. In many cases, intraoral clinical manifestations are similar to those of squamous cell carcinoma and the diagnosis can only be established through biopsy [26, 90]. As can be seen, the differential diagnosis of dental infections and benign or malignant lesions is essential for treatment.

## Conclusion

Overall, lymphomas respond very well to chemotherapy and oral manifestations start regressing from the seventh day of treatment. Therefore, misdiagnosing and applying the wrong treatment, such as dental procedures, may end up delaying the correct diagnosis and worsening the prognosis. A good medical history, detailed clinical and imaging evaluations, and attention to the patient’s signs and symptoms are crucial for the correct diagnosis and appropriate treatment, which in turn can lead to better patient prognosis.

## Figures and Tables

**Figure 1.** Strategy protocols.

Databases	Sensitive search strategy	Results
**Pubmed**	((Oral manifestations [mh] or oral [ti] or mouth [ti] or ‘oral manifestations’ [ti]) and (lymphoma [ti] or Lymphoma [mh]) and (humans [mesh])) not ((‘lymphoma/drug therapy’ [mesh] or ‘lymphoma/therapy’ [mesh]) and (humans [mesh]))	428
**Lilacs**	Oral and (linfoma or lymphoma)	153
**Cochrane library**	#1 MeSH descriptor: [oral manifestations] explode all trees #2 oral or mouth or ‘oral manifestations’ #3 #1 or #2 #4 MeSH descriptor: [signs and symptoms] explode all trees #5 symptom* or manifest* or clinical* #6 #4 or #5 #7 Lymphoma #8 MeSH descriptor: [lymphoma] explode all trees #9 #7 or #8 #10 #9 and #6 and #3 #11 MeSH descriptor: [lymphoma] explode all trees and with qualifiers: [Drug therapy - DT, Therapy - TH] #12 #10 not #11	COCHRANE REVIEWS = 249 COCHARNE TRIALS = 179
**Embase**	#13. ‘mouth’/exp or mouth and (‘disease’/exp or disease) or ‘oral manifestations’:ab,ti or oral:ti or mouth:ti and (‘lymphoma’/exp or lymphoma or lymphoma:ab,ti) not (‘letter’/it or ‘editorial’/it or (‘animal’/exp or ‘animal’:ab,ti not (‘animal’/exp or ‘animal’:ab,ti and (‘human’/exp or ‘human’:ab,ti)))) not (‘drug’/exp or drug and (‘therapy’/exp or therapy) or drug*:ab,ti or chemother*:ab,ti) and clinic*:ab,ti and ([article]/lim or [review]/lim) and ([english]/lim or [portuguese]/lim or [spanish]/lim) and [abstracts]/lim and [embase]/lim and [humans]/lim not (genet*:ab,ti or gene*:ab,ti or protein*:ab,ti or antigen*:ab,ti	447

**Table 1.** Published case reports and retrospective studies.

Author - Year	N of cases	Gender	Age	Diagnosis	Study type
FORMAN - 1970	1	M	25	HL	Case Report
HARMAN - 1971	1	M	57	MF	Case Report
LASKARIS - 1978	1	F	65	MF	Case Report
WRIGHT - 1981	1	M	60	MF	Case Report
HORIUCHI - 1982	87	46 M; 41 F	46 (2 to 82)	HL: 31; ML: 28; DLPD: 20; BL: 05; DWDLL: 03	Retrospective Study
COHEN - 1984	1	F	34	HL	Case Report
BARKER - 1984	7	5 M; 2 F	52 (16 to 84)	LL: 02; LLI: 02; ML: 01; FL: 01; RCC: 01	Retrospective Study
EISENBUD - 1985	6	4M; 2 F	(3 to 16)	BL: 05; DLBCL: 01	Case Report
EVANS - 1986	1	F	52	MF	Case Report
KAUGARS - 1989	1	M	34	DLBCL	Case Report
SODERHOLM - 1989	17	7 M; 10 F	52 (13 to 90)	N/A: 14; BL: 03	Retrospective Study
SPATAFORE - 1989	1	M	46	ML	Case Report
GROOT - 1990	3	3 M	44; 35; 42	BL:01; DML: 02	Case Report
DODD - 1992	3	3 M	43; 40; 46	DLBCL: 03	Case Report
SIROIS - 1993	8	5 M; 3F	62 (49 to 75)	TCL: 08	Case Report
SCULLY - 1993	1	M	15	TCL	Case Report
PILUSO - 1994	2	2 M	26; 37	IL: 02	Case Report
ROSENBERG - 1996	2	1 F; 1 M	75; 61	TCL: 02	Case Report
SHINDOH - 1997	52	29 M; 23 F	54 (5 to 86)	DLL: 23; ML: 05; SNC: 06; FL: 03; MFL: 02; BL: 01; IL: 08; NC: 02; DSC: 02	Retrospective Study
NITTAYANANTA - 1998	2	2 M	26; 25	BL: 02	Case Report
SAVARRIO - 1999	1	M	77	ALCL	Case Report
MORET - 1999	1	M	81	LNC	Case Report
RICHARDS - 2000	2	2 M	49; 61	DLBCL: 01; TCL: 01	Case Report
DE LA FUENTE - 2000	2	2 F	66; 45	MF: 02	Case Report
EPSTEIN - 2001	361	200 M; 161 F	63 (2 to 96)	DLBCL: 137; SCL: 99; PL: 23; IL: 11; BL: 06; HL: 03; NC: 82	Retrospective Study
JAEHNE - 2001	26	6 M; 20 F	66 (48 to 74)	NHL: 26	Retrospective Study
CONTRERAS - 2001	4	2 F; 2 M	56; 73; 34; 55	DLBCL; 04	Case Report
NALLI - 2003	12	9 M; 3 F	45 ( 7 to 82)	N/A: 12	Retrospective Study
WAIN - 2003	1	M	9	MF	Case Report
VAN DER WAAL - 2004	40	24 M; 16 F	46 (3 to 88)	DLBCL: 40	Retrospective Study
RADHAKRISHNAN - 2005	1	M	7	PBL	Case Report
ALBUQUERQUE - 2005	1	F	30	TCL	Case Report
KOJIMA - 2006	1	F	64	MALT	Case Report
OTMANI - 2007	37	31 M; 6 F	7 (2 to 15)	BL: 37	Retrospective Study
JHAM - 2007	1	M	62	DLBCL	Case Report
FARIAS - 2008	1	M	54	DLBCL	Case Report
FREITAS - 2008	1	M	7	BL	Case Report
KEMP - 2008	40	19 M; 21 F	71 (35 to 89)	DLBCL: 23; FL: 06; ENMZL: 05; PL/MM:03; SLL/CLL: 02; TCL: 01	Retrospective Study
KESZLER - 2008	40	23 M; 17 F	49 (3 to 90)	DLBCL: 09; PL: 09; LCPB: 05; BL:06; PBL: 05; FL: 05; LHG: 01	Retrospective Study
DE-MISA - 2008	1	M	72	LyP	Case Report
SANTOS - 2009	1	M	22	DLBCL	Case Report
VILLA - 2009	1	M	57	TCL	Case Report
BEGON - 2010	1	F	34	TCL	Case Report
BORTOLUZZI - 2010	1	M	73	DLBCL	Case Report
RAO - 2010	2	2 M	65; 70	PBL: 02	Case Report
PEREIRA - 2010	2	2 M	4; 24	BL:02	Case Report
BULUT - 2011	1	M	74	DLBCL	Case Report
INCHINGOLO - 2011	1	M	73	NHL	Case Report
BOMBECCARI - 2011	1	M	11	MALT	Case Report
CHI - 2012	1	F	40	DLBCL	Case Report
CHICARELLI - 2012	1	M	45	MCL	Case Report
KIKUCHI - 2012	1	F	61	BL	Case Report
TRIANTAFILLIDOU - 2012	58	32 M; 26 F	45 (8 to 81)	DLBCL: 19; SLL/CLL: 10; MALT: 21; MCL: 04; FL: 02; BL: 01; B-ALL: 01	Retrospective Study
FORTUNA - 2012	1	F	39	PBL	Case Report
GABALI 2013	1	M	11	MALT	Case Report
FERREIRA 2013	1	F	38	TCL	Case Report
PINISETTI -2013	1	F	38	BL	Case Report
SCHWETHA - 2014	1	F	62	NHL	Case Report
MEDEL - 2014	1	M	52	PBL	Case Report
COZZOLINO - 2014	14	4 M; 10 F	68 (45 to 84)	DLBCL: 8; MALT: 4; FL 2	Retrospective Study
RAMANATHAN - 2014	42	25 M; 17 F	48 (2 to 40)	BCL: 17; TCL: 10; BL: 6; NHL: 6; DLBCL: 3	Retrospective Study
OWOSHO - 2014	26	13 M; 13 F	(33 to 88)	DLBCL: 20; MCL: 1; DLBCL/BL :5	Retrospective Study
CORTI - 2015	1	M	39	PBL	Case Report
CUESTAS -2015	1	M	7	BL	Case Report
SAMOON - 2015	1	F	30	PBL	Case Report
PHILIPONE - 2015	47	22 M; 25 F	70.8 (36 to 94)	DLBCL: 13; SLL/CLL:8; FL:6; MALT: 5; PL:5; PBL: 3; MCL: 2; TCL: 2; NHL: 2; BCL: 1	Retrospective Study

HL = Hodgkin’s Lymphoma; MF = Mycosis Fungoides; ML = Malignant lymphoma; DPDL = diffuse poorly differentiated lymphocytic lymphoma; BL = Burkitt’s lymphoma; DWDLL = diffuse well-differentiated lymphocytic lymphoma; LBL = lymphoblastic lymphoma; RCC = reticular Cell Carcinoma; LL = lymphoblastic lymphosarcoma; LLI = lymphocytic lymphosarcoma; FL = follicular lymphoma; DLL = diffuse lymphoblastic lymphoma; DLBCL = diffuse large B-cell lymphoma; N/A = not available; DML = diffuse mixed lymphoma; TCL = T-cell lymphoma; IL = immunoblastic lymphoma; DLCL = diffuse large-cell lymphoma; SNC = small non-cleaved cell malignant lymphoma; NC = Not Classifiable; MFL = mixed malignant follicular lymphoma; DSC = diffuse malignant small-cleaved-cell lymphoma; ALCL = anaplastic large-cell lymphoma; LNC = large non-cleaved cell non-Hodgkin’s lymphoma; NHL = non-Hodgkin’s lymphoma; SCL = small cell lymphoma; PBL = plasmablastic lymphoma; PL =plasmacytoma; MALT = extranodal marginal zone lymphoma of mucosa-associated lymphoid tissue; ENMZL = extranodal marginal zone lymphoma; PL/MM = plasmacytoma/multiple myeloma; SLL/CLL = small lymphocytic lymphoma / chronic lymphocytic leukemia; LHG = high-grade lymphoma B; LyP = lymphomatoid papulosis; B-ALL = acute lymphoblastic leukemia B; MCL = mantle-cell lymphoma; DLBCL/BL: B-cell lymphoma unclassifiable with features intermediate between DLBCL and BL; BCL = B-cell lymphoma.
